# On the Coloration of the Bones by Madder

**Published:** 1842-12

**Authors:** 


*On the Coloration of the Bones by Madder.*—MM. Serres and Doyere have been engaged for upwards of two years in a long course of experiments on the coloration of the bones of living animals by feeding them with madder, and have at length arrived at some very curious and interesting results both with regard to that subject and the mode in which bone is formed. They found that when an animal was fed for a long time on madder, not only were the bones colored of a rose hue, but all the other solids and fluids of the body, with the exception of the tendons and cartilages and the white substance of the brain. In the bones alone they found this color to be permanently fixed, and not removable by maceration, nor by certain chemical solvents of the coloring matter of madder. Maceration, however, removed the coloring matter from all the other tissues. They ascertained, from a chemical investigation, that it was the phosphate of lime which held permanent the coloring matter of madder in the bones. They found that the coloring matter did not penetrate the bones to any depth, but the distribution of the color was remarkable as throwing much light on the true structure of the osseous tissue.

When a long bone colored with madder, is sawed transversely, the colored portion is observed to penetrate but a little depth, forming, as it were, a circle round the bone. When this section is examined by means of a magnifying power of twenty diameters or so, the coloring matter is seen to exist as red points scattered over a white ground. When a power of two or three hundred diameters is used, each point is seen to be in reality a colored circle surrounding a round aperture in the substance of the bone. A longitudinal section of the same bone exhibits the true structure of the bone more particularly; and proves that this rounded aperture seen surrounded by a colored ring in the transverse section, was the section of a minute canal, or capillary vessel, which is more delicate as the animal is older. The colored ring surrounding it was seen to be the walls of this canal, or that part of the bony structure which constituted its walls, and which differed in no respect from the uncolored portion of the bony tissue which surrounded it.

The most exterior layer of coloring matter seen on the outer surface of the bone was found to consist of a colored layer, as thin as that which was seen surrounding each bony capillary vessel in the interior of the bone. The bone then was seen only to be colored at those portions which were in immediate contact with the capillary vessels of its tissues or of the periosteum, and at no other part.

MM. Serres and Doyere next pass to the examination of what it has been the custom of late to describe as the osseous corpuscles, and they show, by the simplest of all experiments, viz. by allowing a drop of oil to come in contact with a slice of bone under the microscope, that these so-called osseous corpuscles, on which so much stress has been laid, are neither more nor less than microscopic deceptions,—are, in fact, cavities or empty cells, which they think are possibly filled with a fluid during life, but not with the circulating fluid, as they they are never colored with madder.

These experimentalists ascertained another important fact. They fed a young pigeon with madder from the 10th of March to the 15th of April, 1840, when they amputated the wing, and found the bones deeply stained with the coloring matter. The madder was then stopped; it was nourished on the ordinary food of these animals; and on the 30th of January, 1841, the other wing was cut off, from the effects of which operation it died. Though this was a young animal, only four months old when it lost its first wing, and though the bones had been exposed to the incessant vital actions for so many months after the madder was stopped, its bones, when it died, presented the very same depth of color as the wing which had been amputated so many months before. MM. Serres and Doyere, therefore, justly infer, that the asserted incessant change or renewal of the molecules is not an essential condition of the living tissues, unless bones are to be ranged among the dead ones.

From these investigations, then, it appears that the staining in the bones by madder is purely a chemical process,—a species of dyeing. That in the colored ring which the naked eye discovers on the long bones, the coloring matter is confined to that portion of the osseous tissue in contact with the periosteum, and that forming the immediate walls of the capillary vessels of the bone. That, as the distance of each capillary vessel from each other is more than double the breadth of the colored layer surrounding each, even in the colored ring the greater portion of the bone is really uncolored, really white. And, lastly, that the dyeing of the bone is influenced by the mode in which the blood is distributed through it; some bones, from the peculiarities of their circulation, being colored from within outwards, otherwise from without inwards.—*Edin. Med. and Surg. Jour.* July 1842, from *Comptes Rendus*, February, 1842.

